# Stimulation of Different Sectors of the Human Dorsal Premotor Cortex Induces a Shift from Reactive to Predictive Action Strategies and Changes in Motor Inhibition: A Dense Transcranial Magnetic Stimulation (TMS) Mapping Study

**DOI:** 10.3390/brainsci11050534

**Published:** 2021-04-24

**Authors:** Luigi Cattaneo, Sara Parmigiani

**Affiliations:** 1 Center for Mind/Brain Sciences (CIMeC), University of Trento, Via delle Regole 101, 38123 Trento, Italy; 2 Department of Neuroscience, Biomedicine and Movement, University of Verona, Piazzale L.A. Scuro 10, 37134 Verona, Italy; 3 Department of Biomedical and Clinical Sciences “L. Sacco”, University of Milan, Via G. B. Grassi 74, 20157 Milano, Italy; sara.parmigiani@unimi.it

**Keywords:** action inhibition, motor control, face movements, transcranial magnetic stimulation, dense sampling mapping

## Abstract

Delayed motor tasks require timely interaction between immobility and action. The neural substrates of these processes probably reside in the premotor and motor circuits; however, fine-grained anatomical/functional information is still lacking. Participants performed a delayed simple reaction task, structured as a ready-set-go sequence, with a fixed, predictable, SET-period. Responses were given with lip movements. During the SET-period, we performed a systematic dense-mapping of the bilateral dorsal premotor region (dPM) by means of single transcranial magnetic stimulation (TMS) pulses on an 18-spot mapping grid, interleaved with sham TMS which served as a baseline. Reaction times (RTs) in TMS trials over each grid spot were compared to RTs in sham trials to build a statistical parametric z-map. The results reveal a rostro-caudal functional gradient in the dPM. TMS of the rostral dPM induced a shift from reactive towards predictive response strategies. TMS of the caudal dPM interfered with the SET-period duration. By means of dense TMS mapping, we have drawn a putative functional map of the role of the dPM during the SET-period. A higher-order rostral component is involved in setting action strategies and a caudal, lower-order, part is probably involved in the inhibitory control of motor output.

## 1. Introduction

### 1.1. The Neural Bases of Immobility Behavior

Behavioral control encompasses a mosaic of different processes, ranging from moving at the right time with the appropriate action to reactively or proactively withholding, postponing, inhibiting, or even changing action plans due to prompted or unprompted incentives [1,2,3,4]. Hence, what is behaviorally described as *not moving* is not simply the lack of movement, instead it requires active and accurate control for shaping the motor strategy for stillness that could lead to initiating and performing subsequent actions when the right time comes or to cancel the preloaded actions in case of need [5,6]. In particular, one behavioral aspect in which the interplay between withholding an action and releasing it is of paramount importance is the so-called SET-period that precedes a delayed cued action. The neural activity in the SET-period of a delayed task is generally considered to be related to action preparation and to keeping a memory trace of the cued action in non-human and human data [7,8]. However, in terms of successful behavior, the SET-period is characterized not only by the need to keep an action ready to be released, but also by the necessity not to move. Hence, at least a part of the SET-period’s neural activity must be inhibitory (see, for instance, [9,10]). In addition to this, the brain’s strategies to cope with an SET-period could in turn be either a bottom-up reactive response to an external imperative GO cue or, whenever the timing of the GO cue is predictable, a top-down, proactive internal estimation of the time of the cue onset. Preventing unwanted anticipated responses usually implies an interplay between inhibition and facilitation of the involved effectors [1,6,11,12,13,14]. At the beginning of the SET-period, the inhibitory information should prevail and, subsequently, a reversal from inhibition to facilitation should overcome when the right time to release the action is coming [9,10]. The neural substrates of this strategic process reside in the premotor–motor circuit and in the frontal cortices for their cortical trait, and in the subthalamic nucleus (STN) for the subcortical part. For the cortical part, there is consistent evidence in monkeys and humans suggesting that the dorsal and medial premotor cortices might be critically involved in these features. Dorsally, the dorsal premotor cortex (dPM) neurons exhibit a rich variety of set-related activity, being time-locked to the ongoing inhibitory behavior [13,15,16,17,18,19,20,21,22]. Medially, the supplementary and pre supplementary motor areas complex (SMA proper and pre-SMA) were together described as the “negative motor area” [4,23,24] and associated with several “negative” or inhibitory motor phenomena in humans [19,25,26]. Of course, there is evidence that SMA proper and pre-SMA impact motor behavior not only in stopping it, but also in initiating it, or even forcing it. Damage to these regions produces different degrees of impairment in motor initiation, from the akinetic pattern of the so called SMA syndrome [27] to utilization behavior and alien hand syndrome, where the impulse to act cannot be stopped far beyond conscious intentions [28]. Finally, also functional magnetic resonance (fMRI) studies pointed at both the dPM and SMA complex as an important node in the fronto-parietal network for response inhibition and control, even in the case of withholding and then cancellation of movements [24,29,30,31]. Concerning the interaction between the cortical trait and the subcortical one, it has been shown that the STN has a key role during conflicting choices decisions and inhibitory control. First, STN activation interacts with the globus pallidus, which in turn suppresses the thalamus and M1 [32]. Taking advantage of transcranial magnetic stimulation (TMS), Coxon and colleagues [33] confirmed a short-interval-intracortical inhibition, which increased in contralateral M1 with respect to the responding hand when subjects successfully inhibited their response. Second, it has been suggested that STN activity is crucial for delaying movements until sufficient information to select the correct option is available [34,35], avoiding impulsive decisions in the frontal cortex. More recently, bilateral deep brain stimulation (DBS) of STN during a STOP-signal task has been repeatedly shown to improve reactive (see, for instance, [36,37,38,39]) and proactive inhibition of upper limb movements [40]. This latter work also showed that bilateral stimulation of STN in Parkinson’s (PD) patients restores a planning strategy for reaching arm movement, generating context-appropriate movements.

### 1.2. A Specific Dorsal Premotor-Primary Motor Circuit Controls Immobility during the SET-Period

In our group, we ran a series of experiments using TMS to investigate the role of the dPM in inhibitory control during the SET-period of delayed motor tasks. First, we demonstrated the existence of direct inhibitory connections between a specific sub-sector of the dPM along the superior frontal sulcus and the primary motor cortex (M1) in baseline conditions, i.e., when the subject was standing voluntarily still [41]. In a subsequent experiment, we showed that such inhibitory connections between dPM and M1 are task specific and are prominent in the middle portion of the SET-period [13]. In a last experiment [42], we demonstrated that dPM stimulation during a GO-NOGO task selectively impaired the capacity to withhold the motor response in NOGO trials. Taken together, our previous results indicated that a specific direct connection between the dPM and M1 mediate the capacity not to move during the set-period when this is required for successful behavior. Despite the growing body of evidence, to date we still lack a fine-grained functional specialization for the control behavior of these areas in healthy humans. Little is known about the gradients concerning their predictive and reactive behavior, and many open questions about the precise cortical involvement in withholding and preparatory phase of instructed actions are still on the table [31,43,44]. 

### 1.3. Dense TMS Mapping and Experimental Protocol

To start filling this gap in knowledge described above, we performed a systematic dense-mapping of the bilateral dorsal and medial premotor cortices by means of TMS during a task requiring healthy participants to perform a delayed lower facial movement, reacting to an imperative external cue after a fixed-time (800 ms) preparatory SET-period. There are two main advantages of our approach. First, the adoption of a dense-mapping stimulation protocol (dense TMS mapping, as defined in [45]). This consists in applying TMS to a broad, evenly distributed grid of stimulation points in a given region of interest, to build a cortical map of the effects of TMS. The outcome of such mapping procedure is a standardized statistical parameter map that allows for comparisons between subjects and therefore to build a population map. The main advantage of the use of TMS dense maps is that the anatomical location of TMS is not decided a priori, therefore avoiding logical short-circuits between the choice of a single area to be stimulated and the anatomical inferences on the function of that area. Using dense grids of TMS targets allows for performing functional mapping of cortical functions that is independent of a priori hypotheses on anatomical localization, resulting in robust spatial information on functional brain maps [46,47,48]. Here, a grid of 18 active TMS spots was drawn on the dPM of each subject with coverage of both hemispheres (nine spots per side). The choice of a bilateral mapping is due to the fact that preparatory inhibitory mechanisms have been shown to be symmetrical between the two hemispheres [49] for upper limb movements, not relying on the right hemisphere only, but more likely on the cooperation between the two hemispheres, as shown in [50]. They compared the inhibitory performance of right-dominant (RPD) and left-dominant (RPD) PD patients and they did not find any difference in either reactive or proactive inhibition between LPD and RPD patients, even though patients were impaired with respect to healthy controls. More recently, analogue results have been found in PD patients in the early stage of the disease, when the disease is unilateral [51]. Second, participants were instructed to respond with the lips as effector, rather than with the upper limb, allowing for the possibility to stimulate a wide range of spots in the lateral and medial premotor cortices with the assurance that those stimuli are not directly addressing the facial M1, thus not impacting on the effector involved in the task. Indeed, M1 representation of the lower face is far from the portion of the cortex we stimulated (see [52] for a detailed review) so that any effects of dPM TMS cannot be attributed to unwanted M1 stimulation. In addition, the lower face has a strong degree of corticalization in motor control, fully comparable to that of the distal upper limb [52]. The experimental measure was Reaction Times (RTs), which have been considered the main outcome to assess the efficiency of processes that occur during motor preparation following a cortical perturbation [53,54,55]. Since we plan to target with TMS a region that is supposedly involved in immobility behavior, we expect to observe a TMS-related shortening of RTs compared to sham TMS, to be interpreted as a reduced capacity to inhibit the motor cortex. The actual results showed a more complex pattern in which TMS of a caudal region produced the expected shortening of RTs and TMS of a rostral region in the dPM produced an overall shift in action strategy, towards predictive behavior. Moreover, investigating the effects of TMS on the capacity not to move during the SET-period, as we performed here, could not only be of paramount importance in healthy subjects, but it can also be a relatively easy measure for comparing them with clinical populations, such as PD, attention-deficit hyperactivity disorder (ADHD), substance addiction or pathological gambling. It could enable us to disentangling differential cortical contribution from motor preparation and action inhibition, as well as to tell apart normal predictive and reactive behaviors.

## 2. Materials and Methods

### 2.1. Participants

Seventeen healthy participants took part in this study (9 female, age range 19–35). They all provided informed consent and were screened for any contraindication to TMS [56]. They were right-handed, as assessed by the Edinburgh handedness inventory [57], with no previous history of neurological or psychiatric disorders. The study was conducted in the Department of Neuroscience, Biomedicine and Movement of the University of Verona, with procedures compliant with the revised Helsinki declaration (2009) and approved by the Department’ ethical committee.

### 2.2. Experimental Protocol

**Figure 1:**
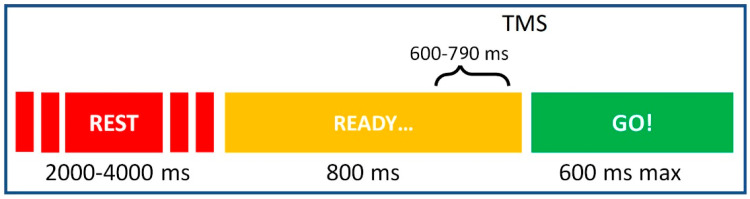
Schematic representation of each single trial of the experiment and its time course. The inter-trial interval lasted from 2000 to 4000 ms, then the READY cue appeared for 800 ms, identifying the SET-period requiring participants to be ready but not to move. In the last 200 ms of the SET-period, TMS was randomly delivered [13], with a jitter applied to avoid the expectation of the magnetic stimuli (no effect of the jitter on the performance). Then, the GO signal informed participants to lift the stick with their mouth as quickly as possible, in a 600 ms range for a possible response.

Participants were sitting comfortably, with the head placed on a chin rest secured to a table and an additional lateral head-constraint, which assured head stability, prevention of fatigue and minimal unrelated movements during the execution of the orofacial action. They were asked to rest with eyes open, wearing earplugs for the whole experiment, and to hold the wood stick between their lips. The experimental protocol consisted in a simple “warned” or “delayed” reaction time task [9,10,58]. Single-pulse TMS were delivered in an event-related timing, ranging from 600 to 790 after the SET-period onset. Stimuli were presented with Matlab software, on a 75 Hz (1680 × 1050 resolution) 20″ monitor, at 45 cm of distance from the participant’s eyes. Each trial (Figure 1) started with an SET-period of a predictable duration (800 ms) indicated with a green fixation cross, during which participants had to stay still and be READY, followed by a GO signal. The GO signal lasted up to 600 ms and was indicated with a circle in the middle of the screen. After the response (lifting a stick with their lips as fast as possible from a starting to an ending point) was given, the corresponding reaction time was displayed on the screen, serving as feedback of individual performance. Then, the variable inter-trial interval occurred (with a duration ranging from 2000 to 4000 ms), instructing participants to REST. Any anticipation of the response prior to the GO was considered an error. It should be noted that, given the nature of the SET-period, the onset of the GO signal was entirely predictable throughout the experiment.

### 2.3. Apparatus for Lip Response Collection

**Figure 2:**
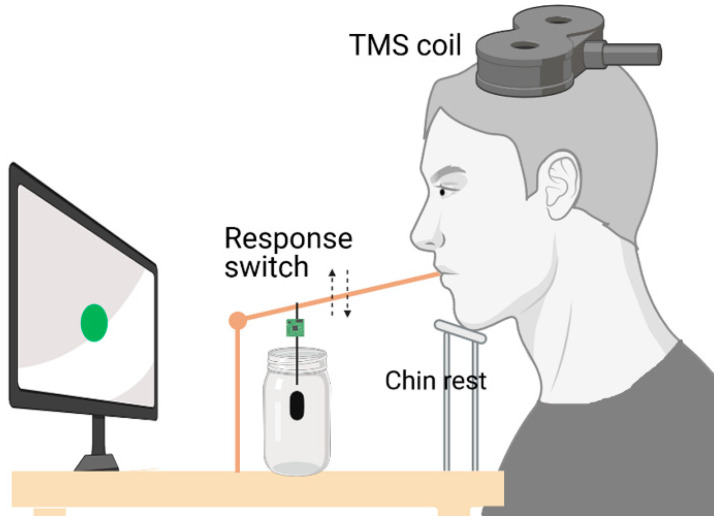
Schematic illustration of the experimental setup.

All participants were required to hold a wood stick with their lips devoted to collect the motor responses, as reported in our previous works [13,41,42]. At the end of the stick there was a 15 g weight, with the aim to stabilize the apparatus and make the return to the starting point easier (Figure 2). The proper motor response consisted in lifting the stick from a starting point until the end position was met, after which no more lifting was possible. At the instant when the end position was reached, a circuit was closed providing a +5 V square-wave output. The output signal was transmitted to a PC via the serial port by the Matlab software. After each trial, response time (RespT) was displayed on the screen to give online feedback to the participants.

### 2.4. Building of the TMS Dense Grid

**Figure 3:**
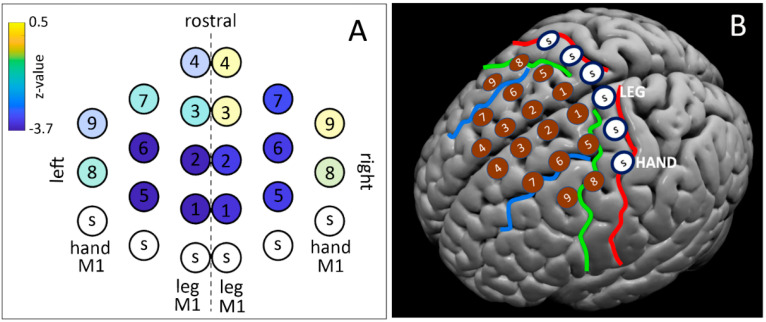
(**A**) Grid spots of z-values of the population analysis on RTs (negative z-values indicate shorter RTs in TMS compared to sham). Active TMS spots are indicated with a number. Sham TMS spots are indicated with the letter ‘S’. (**B**) Projection of the cortical targets on a brain surface template, with the eighteen effective spots and the sham (S) spots. Red: Central Sulcus, Green: Precentral Sulcus, Blue: Superior Frontal Sulcus. LEG indicates the leg motor hotspot. HAND indicates the hand motor hotspot.

TMS was delivered using a dense grid protocol [45] to 9 points located in the premotor and supplementary motor areas for both hemispheres, resulting in a total of 18 points stimulated. They were outlined in the medial–lateral axis and in the rostro–caudal axis starting from the functional localization of hand and foot representations in the M1 in both hemispheres (see Figure 3). To draw a grid on the scalp’s subject, the hand and foot representations were defined as the spot on the scalp where the larger MEP from *first dorsal interosseus* (hand) and the *tibialis* muscle (foot) could be obtained with the lowest intensity. Then, a spot halfway between the hand and foot hotspots was marked. These 3 spots were used as targets for ineffective (sham) TMS (white spots with ‘s’ label in Figure 3B). The remaining spots were found by moving 1.5 cm rostrally from the 3 sham spots. The full geometry of the grid is illustrated in Figure 3, together with a putative projection of the cortical targets on a brain surface template. 

### 2.5. TMS

Magnetic stimulation was achieved by means of a biphasic magnetic stimulator (Magstim rapid) connected to a figure-of-eight coil with 70 mm windings oriented perpendicularly to the midline with the handle pointing with a medio-lateral orientation of the induced current, and the coil adjusted according to the stimulated point. Stimulation intensity was set at the 110% of the resting motor threshold (RMT) for the intrinsic hand muscle first dorsal interosseus. In our former study [13], we described an inhibitory activity of the dorsal premotor cortex on ipsilateral mouth-related M1 during the SET-period, which was found throughout the SET-period, peaking in its mid-portion, invariantly with respect to the duration of the SET-period. According to our model, the second half of the SET-period is characterized by the interaction between the inhibitory and excitatory processes. We therefore applied in the present study single-pulse TMS to evenly cover the later part of the SET-period. To avoid expectation of TMS pulses, the stimuli were delivered in each trial jittered from 600 to 790 after the SET-period onset according to a square-wave distribution.

### 2.6. Data Analysis

All analyses were performed with the MATLAB software, and the full script that was used for data analysis, together with the full dataset, is publicly available at the page https://osf.io/xcjb3/ (accessed on 23 March 2021) of the Open Science Framework repository. We collected a total of 16 trials per each of the TMS spots. RTs were taken with reference to the GO signal. Therefore, negative RTs corresponded to anticipatory responses. Responses given before TMS were excluded from analysis (overall 1.24% of trials). We planned to exclude any response given after 1000 ms from the GO signal. For any further analysis, the trials in sham TMS spots were pooled together, irrespective of which spot they were obtained from. Sham trials were therefore a total of 92 trials per subject. The main analysis was carried out with RTs as the dependent variable. The main analytical strategy was to perform a within-subject analysis on single trials for each TMS spot compared to sham. The resulting statistical parameters characterizing each TMS spot in each participant were then merged in a population analysis that was used to build the population scalp map of TMS functional effects.

First, we compared within single subjects and, for each TMS spot, the trials in which effective TMS was delivered with the trials in which sham TMS was delivered by means of Mann–Whitney’s U test (MWU). Each MWU produces an output characterized by a statistical parameter referred to as ‘z’, standardized and comparable between subjects. A negative value of z associated with a single TMS spot indicates that in that subject, the RTs associated with TMS on that spot are shorter than the RTs in the sham condition. Vice-versa, positive z-values indicate that effective TMS on that spot is associated with longer RTs than sham TMS. Ultimately, every subject was characterized by 18 z-values, one for each TMS spot, that indicated the relative effect of TMS on that spot compared to sham.

Then, we performed population analysis, by a single-sample MWU, comparing the z-values of the population in each of the 18 TMS spots against the null hypothesis of a mean value = 0. The resulting statistics informed us for each of the effective TMS spots whether the population had individual z-values that were significantly swayed on each side of the 0 value. In this way, the data of the whole population were reduced to a set of 18 z-values that expressed the population’s tendency to perform with faster or slower RTs in the real TMS compared to the sham TMS in each of the 18 effective TMS spots. Such z-values were then used to build the z-map of the scalp surface. For statistical significance, the threshold for the *p*-value associated with each of the spots in the z-map was corrected for 18 multiple comparisons and therefore set to *p* = 0.0027. Note that negative z values indicate that TMS over a specific spot produced trials with shorter RTs than sham TMS.

### 2.7. Post-Hoc Data Analysis on Trials Displaying Predictive Behavior

The distribution of RTs in the population appeared to be biphasic, with an early peak due to predictive, anticipatory behavior and a second peak due to reactive behavior (Figure 3). In the light of this finding, we decided to perform a post-hoc mapping analysis to investigate the effect of TMS on the relative relying of the participant on predictive or on reactive behavior. To do so, we classified each trial as predictive or reactive according to whether the response was given before or after 130 ms, based on the RT distribution shown in Figure 3. We then devised a score that is the ratio between reactive trials and total trials, which was calculated for each participant and each spot. This index, named hereafter ‘reactive index’ is distributed between 0 (no reactive trials, only predictive trials) and 1 (only reactive trials, no predictive trials). We then performed a population analysis, comparing the reactive indexes from each spot to the reactive indexes in the sham condition, by means of MWU. The result was a z-value for each of the effective TMS spots that we used to build a z-map, similar to that constructed for the RT results. For statistical significance, the threshold for the *p*-value associated with each of the spots in the z-map was corrected for 18 multiple comparisons and therefore set to *p* = 0.0027. Note that negative z values indicate that TMS over a specific spot was associated with more predictive trials than sham.

### 2.8. Post-Hoc Data Analysis on Anticipation Errors

To understand whether the decrease in RTs, and the shift to a predictive strategy following TMS, was also associated with increased anticipation errors, we performed a mapping analysis identical to that described in the previous step (post-hoc data analysis on trials displaying predictive behavior) but classifying trials as correct (RT >= 0 ms) or as anticipation errors (RT < 0 ms). We then devised a score that is the ratio between anticipation error trials and total trials, which was calculated for each participant and each spot. This index is distributed between 0 (all trials are anticipation errors) and 1 (no trial is an anticipation error). We then performed a population analysis, comparing the error indices from each spot to the reactive indexes in the sham condition, by means of MWU. The result was a z-value for each of the effective TMS spots that we used to build a z-map, like that constructed for the RT results. For statistical significance, the threshold for the *p*-value associated with each of the spots in the z-map was corrected for 18 multiple comparisons and therefore set to *p* = 0.0027. Note that negative z values indicate that TMS over a specific spot produced more anticipation errors than sham.

## 3. Results

None of the subjects complained of any short- or long-term side effects of TMS. In particular, none reported seizures, syncope, headache, or hearing impairment. No trial was discarded for RT exceeding 1000 ms. The full dataset of single-trial data is publicly available at the page https://osf.io/xcjb3/ (accessed on 23 March 2021) of the Open Science Framework repository.

The main analysis showed several TMS spots in which the RTs were shortened compared to sham TMS, and no spot in which they were increased. The full statistical results are shown in Table 1.

The scalp map of the functional effects of TMS is shown in Figure 3. The TMS spots that induced a significant shortening of RTs were clustered in a dorso-caudal region of the dorsal premotor cortex, bilaterally, with an almost symmetrical pattern between right and left.

**Figure 4:**
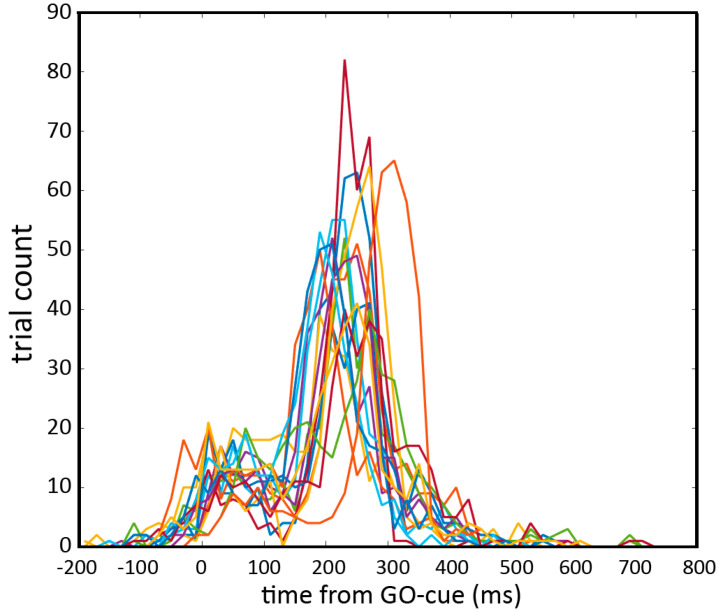
Distribution of reaction times (RTs) from all trials, in the whole population (*n* = 17). Each colored line represents a single subject. Biphasic distribution is visible with a first peak in the 0–130 ms range and a second peak in the 131–500 ms range. Note that negative RTs indicate responses given before the GO cue.

A descriptive analysis of the distribution of RTs indicated a global biphasic distribution, which we interpreted as an expression of anticipatory, predictive behavior (early peak) and a second peak due to reactive behavior, illustrated in Figure 4. Predictive adjustments could also be ascribed to the, more or less implicit, learning that GO signal has a fixed timing, in this sense becoming totally predictable by the participants. The arbitrary delimitation between reactive and predictive behavior was set at 130 ms from the GO signal. 

**Figure 5:**
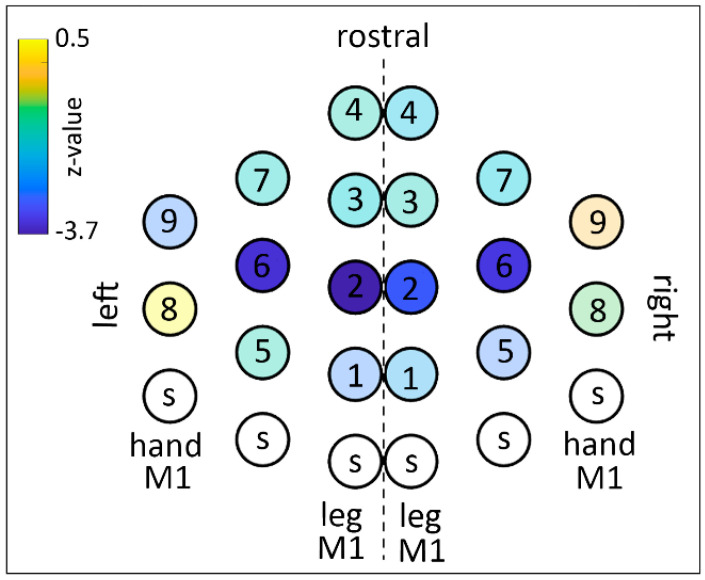
Grid spots of z-values of the population analysis on the ratio between predictive trials to reactive trials. Conventions as in Figure 3. Negative z-values indicate an increased number of predictive trials in TMS compared to sham.

**Figure 6:**
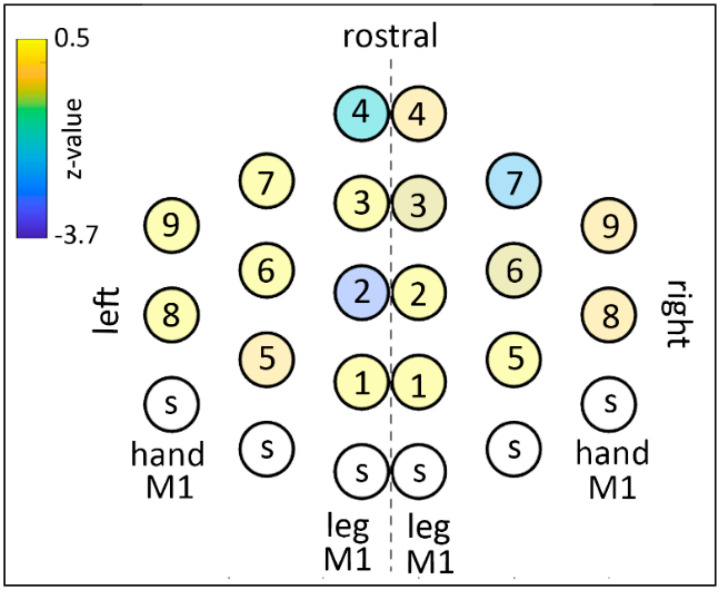
Grid spots of z-values of the population analysis on anticipation errors. Conventions as in Figure 3. Negative z-values indicate an increased number of anticipatory errors in TMS compared to sham.

The mapping analysis to assess the effect of TMS on the ratio between predictive and reactive behavior showed that TMS induced a general shift towards predictive behavior as indicated by a decrease in the ‘reactive index’. This effect reached statistical difference in four symmetrical points in the mid portion of the superior frontal gyrus bilaterally. The map is shown in Figure 5 and the statistical results are shown in Table 2. The mapping analysis to assess the effect of TMS on the occurrence of anticipation errors showed that TMS did not produce significant changes in the number of anticipation errors. The map is shown in Figure 6 and the statistical results are shown in Table 3.

## 4. Discussion

The main aim of the present study was to assess a topographic distribution of control and inhibitory functions in the dorsal and medial premotor cortices of healthy volunteers performing an instructed action with a facial effector. We applied a dense TMS mapping protocol [45] during the 800 ms lasting preparatory phase of a delayed reaction time task [13,58]. Our findings show that the stimulation of a large mid-portion of the dPM induced a significant shortening of RTs. The effective spots clustered bilaterally along the line of the SFG, with an almost symmetrical pattern between right and left (Figure 3). 

A following observation showed that in all participants RTs presented a clear biphasic distribution, unveiling two possible behavioral strategies (Figure 4) that coexisted in all participants. RTs in the early peak are attributed to a predictive behavior because they are significantly shorter than any possible voluntary reactive response to the GO signal. In predictive trials, the subject calculated the timing of the predictable GO signal and produced a motor response without waiting for the GO signal’s occurrence. RTs in the second peak are, on the contrary, likely due to reactive behavior, in which the subject waited for the GO signal and responded to it. 

A further analysis investigated the effect of TMS on the distribution of predictive vs. reactive behavior. We found that a sub-group of the TMS spots that produced shortened RTs also produced a significant shift towards predictive behavior, thus causing the shortening of RTs. A final post-hoc analysis indicated that the shortening of RTs and the shift towards a predictive strategy was not accompanied by an increase in anticipation errors and therefore did not worsen overall performance. Summing up, TMS did not alter behavior in terms of efficiency but rather did so in terms of change of strategy.

### 4.1. A Shift from Reactive to Predictive Behavior

Participants were asked to react to imperative external cues, the READY and the GO cues, and deal with a completely predictable internal estimation of the time between these two cues. Therefore, for a successful performance, this promptness should develop in combination with the internal estimation of the time of the cue onset. Indeed, the disruption of inhibitory cortical nodes reacting to external cues, which leads to a shortening of RTs and to an increase in anticipatory errors, is not the only possible explanation of our results. One may argue that our stimulation interferes with the internal time estimation ability. It is indeed possible that here we are witness to two distinct but intertwined strategies: the preparatory inhibition of a preloaded action and the internal time estimation of the SET-period. Our task can be solved in a proactively, top-down manner, independently from the actual presence of GO signal, or reactively, relying on bottom-up processes, in response to the visual cues. It should be noted that the two strategies are mutually incompatible, and that each strategy has a clear behavioral signature in the RT (Figure 4).

We interpret the current results in terms of local specialization of the dPM for reactive behavior. We hypothesize that, if the dPM is causally needed to carry out reactive behavior, TMS over the dPM during the SET-period has weakened the instantaneous propensity to use the reactive strategy and therefore allowed for a predictive strategy to be carried out. It is likely that such predictive strategy is on the contrary carried out by other portions of the premotor cortex, i.e., the medial premotor cortex or SMA.

There is consistent evidence in the literature showing that during tasks requiring a predictive response, the premotor areas of the medial wall, SMA and pre-SMA contain ramping signals that constitute the neural bases of time prediction (for a review, see [59]). The SMA is therefore strongly and causally involved in predictive behavior (i.e., estimate the time to the GO signal and respond before the GO signal has been processed by the motor system). On the contrary, the dPM is commonly associated with rule-dependent sensorimotor associations, which are at the basis of the reactive behavior (i.e., wait for the GO signal and react to it) as reviewed in [60]. We therefore interpret the current results as a neural imbalance between the dPM and the SMA, induced by TMS, resulting in an imbalance between predictive and reactive behavior. Another key concept, according to which the lateral and medial premotor cortices have been categorized, regards this framework: externally cued actions (represented in the dPM) versus internally generated actions (represented in the SMA). Moreover, the external/internal action framework fits well with the current data. The reactive strategy is, by definition, an externally cued, exogenous behavior. The predictive strategy is fully generated in the subject’s motor system, without any need of sensory input. In fact, most responses given through the predictive strategy occur well before the sensory information on the GO signal has reached the motor system and are therefore entirely independent of external information.

It is worth noting that, according to our interpretation, the interval between TMS and the mouth responses given in the predictive modality is actually very short, being potentially comprised between 0 and 330 ms. It could be argued that such a short time is not enough to reset the strategy of the single trial from reactive to predictive, especially because the predictive strategy requires the estimation of the set-period from its beginning, well before TMS is applied. We hypothesize that for the TMS-induced switch to be possible, both strategies, predictive and reactive are running simultaneously in every trial. The concept of parallel processing in the motor system has been proposed previously, namely, by the *biased selection* model by Cisek and Kalaska [61]. Subsequently, several evidences speak for the simultaneous coexistence of different potential actions up to the motor output in several domains of action [62,63]. It is therefore not unlikely that the two motor strategies, predictive and reactive, might be simultaneously present and trial-by-trial biases shift the actual behavior from one to the other.

### 4.2. A Caudal-Rostral Specialization in the dPM

The main aim of the present work was to assess the function role of the dPM in preparatory inhibition. This has been the subject of considerable debate, since several markers of motor inhibition can be observed during the period preceding a voluntary movement, both in response selection and response initiation [2,22,64]. We learned from studies addressing the direct stimulation of the primary motor cortex that the decrease in MEP amplitude during the SET-period is reflecting cortico-spinal pathway inhibition, revealing a suppression component of action preparation [10,11]. This modulation suggests that inhibitory processes occur in parallel with the activation mechanisms and indicates that optimal time preparation is accompanied by a suppression in activity of the cortico-spinal tract. Some lines of research support the idea that inhibitory processes in dPM are recruited in parallel with facilitating preparatory activity [2], generating inhibitory information targeting the M1, to prevent premature movements [42,65]. 

In our former work [13,41,42], we provided strong evidence of the direct role of the dPM in inhibitory control of M1 and of corticospinal output (see introduction). In the present work, by applying TMS in the phase of the task when dPM actually exerts the inhibitory control over M1 (the SET-period), we expected to interfere with the inhibitory process. We expected such interference to become evident behaviorally as a reduced immobility, namely, as a shortening of reaction times. The results confirm this strong tendency, though a part of the variance in RTs was explained by the shift towards a predictive strategy. In the rostral spots (Spots 2 and 6), the presence of the strategy shift effect could mask a simultaneous effect on RTs. Only a further differential analysis on the effects of TMS on reactive and predictive trials separately could potentially disentangle the two effects, but this was not possible due to the small and variable number of trials in each condition. However, thanks to the dense mapping procedure, we could distinguish in the dPM region a caudal region (Spots 1 and 5 bilaterally) which showed only shortening of RTs, without a significant strategy shift, distinct from the rostral region (Spots 2 and 6 bilaterally) in which the main effect of TMS is that of a strategy shift. We hypothesize that this distinction witnesses a caudo-rostral gradient within the dPM with the caudal portion directly involved in the control of M1 and the rostral part involved in higher-order processes such as response strategy. This hypothesis is supported by several data in the literature that indicate a hierarchical caudal-rostral specialization gradient in the primate dPM, in which the caudal part (F2 sector or dPM-proper codes conditional sensorimotor associations and has direct connections to M1—see [61,66] for a review) and the rostral part have higher-order functions, not related to direct action execution [67,68,69]. The latter, caudal portion is likely to be corresponding to the dPM region involved in the direct inhibitory control described in our previous works [13,41,42]. Although they do not assess proactive inhibitory control, some human’s invasive data could also suggest this specialization gradient. Mattia and colleagues [70] recorded from intracranial electrodes in epileptic patients that underwent presurgical evaluation, showing that M1 and dPM were more involved in reactive inhibitory control than more anterior prefrontal regions.

### 4.3. Limitation of the Present Study

The present study displays some limitations. The first of such limitations is the fact that it was not possible to use individual magnetic resonance imaging (MRIs). Although a further study relying on individual MRIs will be beneficial to improve our anatomical resolution and details, here, we partially overcome this limitation using the dense grid protocol, covering bilaterally the dPMC. Stimulation points were localized starting from the functional identification of hand and foot representations in the M1 in both hemispheres and then a comparable grid on each scalp’s subject was marked, a putative projection of the cortical targets on a brain surface template was established, and stimulation intensity was adjusted accordingly. Therefore, although it is necessary to refer to putative cortical targets, the fine functional identification and constant online control of the spots’ location in the surface template and with respect to the selected hotspots in M1, along with the dense grid protocol, assured a high level of anatomical accuracy, demonstrating that this approach also is easily feasible in conditions in which the individual MRIs are not available. 

The second limitation is the fact that the unveiling of reactive/predictive strategies was somehow serendipitous. Indeed, the main aim of the present study was to investigate the role of different sectors of the dPMC in action inhibition, with site of stimulation as the sole variable. We found that a different portion of the cortex not only is involved in a differential fashion in the inhibitory control and in the release of the preloaded action, but is also engaged differently in the shifting from reactive to predictive action strategies. However, such predictive shifts are possible since participants applied the form of proactive/predictive adjustments while they predict the outcome of the GO signal. This form of adjustments, however, might have some differences with respect to task in which subjects are aware of acting in different contexts and changing their behavioral strategy accordingly. For instance, in [71], proactive inhibition was evaluated by comparing RTs and MT of GO trials in a GO/NOGO task with those measured during the execution of the same movements in the context of a simple RT task (GO-only trial). This phenomenon, named *context effect*, represents an optimization of the motor strategy in the two different contexts, [71]. Future studies need to address this issue, with a task designed to distinguish between reactive and predictive strategies, in which subjects are aware of acting in different contexts and changing their behavioral strategy accordingly.

## 5. Conclusions

The current study contributes to composing a detailed functional cartography of the healthy premotor cortices during the performance of a simple movement to be released as fast as possible when the right time comes. Taking advantage of a dense TMS spatial mapping approach, with the assurance that our stimulation is not directly addressing the lower facial M1—and thus not impacting the effector involved in the task—our stimulation revealed a causal and different involvement of the subdivision of dPM in controlling lower face movements. A rostral portion of the dPM represents a higher-order station where reactive action strategies are defined. Conversely, a more caudal part of the dPM has no role in instantiating action strategies but is likely to have a direct task-dependent inhibitory influence on M1.

## Figures and Tables

**Table 1:** Results of main analyses on RTs. Z-values (*p*-value) for each spot are reported. The correspondent map including grid-spot numbering is shown in Figure 2. Data exceeding the significance threshold (*p* = 0.0027) are shown in bold.

Grid Spot	Right Hemisphere	Left Hemisphere
1	**−3.43 (0.0006)**	**−3.62 (0.0003)**
2	**−3.29 (0.001)**	**−3.62 (0.0003)**
3	0.4 (0.69)	−1.63 (0.1)
4	0.5 (0.62)	−2.82 (0.005)
5	**−3.29 (0.001)**	**−3.62 (0.0003)**
6	**−3.34 (0.001)**	**−3.62 (0.0003)**
7	**−3.15 (0.002)**	**−1.59 (0.11)**
8	−0.78 (0.43)	−1.4 (0.16)
9	0.36 (0.72)	−2.72 (0.01)

**Table 2:** Analysis on the prevalence of predictive behavior compared to reactive behavior. Figure Z-values (*p*-value) for each spot are shown. The correspondent map including grid-spot numbering is shown in Figure 3. Data exceeding the significance threshold (*p* = 0.0027) are shown in bold.

Grid Spot	Right Hemisphere	Left Hemisphere
1	−2.26 (0.0238)	−2.66 (0.0079)
2	**−3.01 (0.0026)**	**−4.35 (0.0001)**
3	−1.49 (0.14)	−1.67 (0.09)
4	−1.92 (0.05)	−1.42 (0.157)
5	−2.69 (0.0071)	−1.36 (0.1724)
6	**−3.39 (0.001)**	**−3.46 (0.0005)**
7	−1.81 (0.07)	−1.56 (0.12)
8	−0.99 (0.32)	1.13 (0.26)
9	−0.28 (0.78)	−2.68 (0.01)

**Table 3:** Analysis on the prevalence of anticipation errors compared to reactive behavior. Figure Z-values (*p*-value) for each spot are shown. The correspondent map including grid-spot numbering is shown in Figure 3. Data exceeding the significance threshold (*p* = 0.0027) are shown in bold.

Grid Spot	Right Hemisphere	Left Hemisphere
1	1.1 (0.271)	1.1 (0.271)
2	1.1 (0.271)	−2.79 (0.0052)
3	−0.56 (0.58)	1.1 (0.27)
4	0 (1)	−1.67 (0.095)
5	0.53 (0.5966)	0 (1)
6	−0.56 (0.576)	1.68 (0.0927)
7	−2.24 (0.025)	2.27 (0.02)
8	0 (1)	1.68 (0.09)
9	0 (1)	1.1 (0.27)
